# Secular Trends in the Size and Shape of the Scapula among the Portuguese between the 19th and the 21st Centuries

**DOI:** 10.3390/biology12070928

**Published:** 2023-06-28

**Authors:** Ruben Maranho, Maria Teresa Ferreira, Francisco Curate

**Affiliations:** 1 Laboratory of Forensic Anthropology, Department of Life Sciences, University of Coimbra, Calçada Martim de Freitas, 3000-456 Coimbra, Portugal; ruben.maranho@student.uc.pt (R.M.); mferreira@uc.pt (M.T.F.); 2 Centre for Functional Ecology, Department of Life Sciences, University of Coimbra, Calçada Martim de Freitas, 3000-456 Coimbra, Portugal; 3 Research Centre for Anthropology and Health (CIAS), Department of Life Sciences, Faculty of Sciences and Technology, University of Coimbra, Rua Arco da Traição 7, 3000-056 Coimbra, Portugal

**Keywords:** secular changes, human scapula, geometric morphometrics, landmarks, semilandmarks

## Abstract

**Simple Summary:**

The human body experiences long-term changes mostly associated with environmental conditions, and generational trends for stature or age at menarche are well-known and studied. That is not the case regarding potential anatomical modifications with time in the human scapula. Accordingly, this study intended to assess size and shape diachronic changes in two Portuguese-reference skeletal collections. Results show that scapular shape and size variation in females is minimal to nonexistent, while in males, an unambiguous decline was detected with time of scapular size. Higher standards of living, including better nutrition and universal healthcare, are associated with an increase in height but also with a slender body—this general trend is possibly related to the scapular size decline with time in Portuguese males.

**Abstract:**

Potential secular changes in the human scapula are fundamentally unbeknownst, with most of the preceding anatomical studies focusing on long-term changes in the long bones and the skull. As such, the cardinal purpose of this study pertains to the evaluation of secular trends on the shape and size of the scapula in a time period spanning from the 19th to the early 21st centuries. The study sample included 211 individuals (100 males and 111 females) from the Coimbra Identified Skeletal Collection and the 21st Century Identified Skeletal Collection. The size and shape of the scapula were evaluated using geometric morphometrics. Results show secular changes over a relatively short period of time in both the shape and size of the scapula in Portuguese nationals. Shape changes were observed in both sexes but expressed minimally, while a significant negative trend in the size of the scapula was detected in males. Scapular size decrement in males conceivably echoes general trends of the overall anatomy towards a narrower body associated with higher standards of living that include enhanced nutrition and universal healthcare, among other factors.

## 1. Introduction

Secular trends refer to biological changes that take place over decades or generations [1]. The best-documented secular trends are the increment in stature and the anticipation of age at menarche [2]. Long-term changes can be precipitated by the restraint of factors that inhibit growth [3]. These are attributed to the improvement of living conditions that started in the late 19th and early 20th centuries, especially in the more industrialized Western countries [4], e.g., dietary improvement—with lower or no periods of caloric deficit—better sanitation, reduction of infant mortality rates due to medical advances and public health policies, as well as rising socio-economic status [5,6,7].

Although environmental factors are acquainted as the precipitating factor for secular changes, their mechanism is not fully understood [5,8]. The primary mechanism cited is phenotypic plasticity, as environmental changes can alter gene expression without altering the DNA sequence and be passed to the next generations [9,10]. However, phenotypic plasticity is probably not responsible for all changes [5]. The increase in heterozygosity, derived from the gene flow caused by migrations, is also involved in long-term changes [8,10,11]. Although it is a small interval of time for a genetic change, selection cannot be excluded as rapid evolution has been documented for several organisms, including humans [12,13,14,15]. Other authors consider that reduced infant mortality is strongly related to secular change since it contributed to the exponential rise of population, influencing the rate and range of less prevalent phenotypic variations [6,16,17].

The global trend shows an increase in height and the length of some long bones—mainly those directly associated with stature—but not all populations display identical patterns of growth [18]. Therefore, secular trends can be classified as positive if they demonstrate an increase in size [19,20], absent if the population does not present any changes [21], or negative if size diminishes [18,20,22,23,24]. Different coexisting trends can be discerned within any given population [6,10,25]. Secular changes have been identified in distinct skeletal elements, such as the cranium [5,6,18,26,27], the long bones (femur, tibia, radius, and humerus) [20,25,28,29,30], the clavicle [31], and the pelvis [24,32]. The scapula has been extensively utilized for assessing parameters such as biological sex and stature [33,34,35,36], but its analysis in terms of secular changes has been lacking.

Most of the previous research studies regarding secular changes in the skeleton are exclusively based on traditional morphometrics and size differences between the long bones and the skull. In this study, a geometric morphometrics (GM) approach to time-related variation in the scapula will be favored. GM is a compilation of techniques that convey a mathematical description of biological shape, enabling the analysis of structures with curves and protuberances that were disregarded by conventional morphological methods [37]. GM allows for describing, analyzing, and interpreting shapes and their variation, evaluating anatomical differences between groups with negligible subjectivity [38,39,40]. GM utilizes Cartesian coordinates, or landmarks, that retain shape information [41]. Landmarks can be defined as discrete homologous points of correspondence among individuals [42]. Regrettably, landmark analyses cannot quantify all morphological structures, such as curves and surfaces. Semilandmarks allow quantifying two three-dimensional homologous curves and surfaces and analyzing them simultaneously with traditional landmarks [38,43,44].

This study is based on two Portuguese-reference skeletal collections: the Coimbra Identified Skeletal Collection and the 21st Century Identified Skeletal Collection. The main objectives of this research pertain to the evaluation of potential secular trends in the shape and size of the scapula from the 19th to the early 21st centuries, the description of size and shape differences between the skeletal collections and the patterns of secular change in each biological sex, using geometric morphometrics techniques on two-dimensional photographic images of the scapula.

## 2. Materials and Methods

This research was implemented in two Portuguese-reference skeletal collections curated at the Department of Life Sciences (DCV) of the University of Coimbra: the Coimbra Identified Skeletal Collection (CISC) and the 21st Century Identified Skeletal Collection (CEI/XXI) [45,46,47,48,49]. The CISC comprises 505 individuals [47,48]. The skeletonized remains, originating from unclaimed bodies, were retrieved from the Cemitério Municipal da Conchada in Coimbra [47,48]. All individuals were born between 1817 and 1924 and died between 1904 and 1938, and their ages at death ranged from 7 to 96 years old [47,48]. The CEI/XXI includes 302 adult individuals that died between 1982 and 2012 that stem from the Cemitério dos Capuchos in Santarém [45,46,48]. The collection includes unclaimed remains of the deceased beyond the legal period [45,46] and mostly elderly individuals [46]. Both collections possess documentation with biographical information for each individual, including age at death and assigned sex at birth [45].

The study sample is composed of the left scapulae of 211 individuals. The CISC subsample encompasses 80 individuals (40 females and 40 males), while the CEI/XXI comprises 131 individuals (71 females and 60 males). The age at death ranges from 17 to 98 years old for females and 19 to 96 years old for males (Table 1). Years of birth range from 1824 to 1918 for CISC individuals (1824–1917 for females and 1844–1918 for males), and years of death are between 1910 and 1938 (1910–1936 for females and 1910–1938 for males) (Table 2 and Table 3). For CEI/XXI individuals, the years of birth range between 1909 and 1981 (1909–1981 for females and 1909–1955 for males), and the years of death are between 1996 and 2012 (1996–2012 for females and 1997–2011 for males).

Only complete scapulae were used in this study. Scapulae presenting pathologies or gross taphonomic alterations were excluded. The assigned sex at birth (i.e., biological sex), age at death, and years of birth and death for each individual were obtained from the documentary sources of each collection [45,46,47].

### Data Collection: Landmarks and Semilandmarks

Data collection procedures are the same as in Maranho et al. [49]. All bones were positioned with the dorsal surface upwards and photographed with a Canon EOS 70D with a Macro lens (50 mm f/2.5) located at a distance of 50 cm. The camera was fitted in a fixed tripod. The position of the scapula was standardized by placing them on an osteometric board with graph paper, with the inferior part of the glenoid fossa and a point on the lateral border touching the vertical surface of the board. The focus of the camera was on a marked spot in the graph paper.

**Figure 1:**
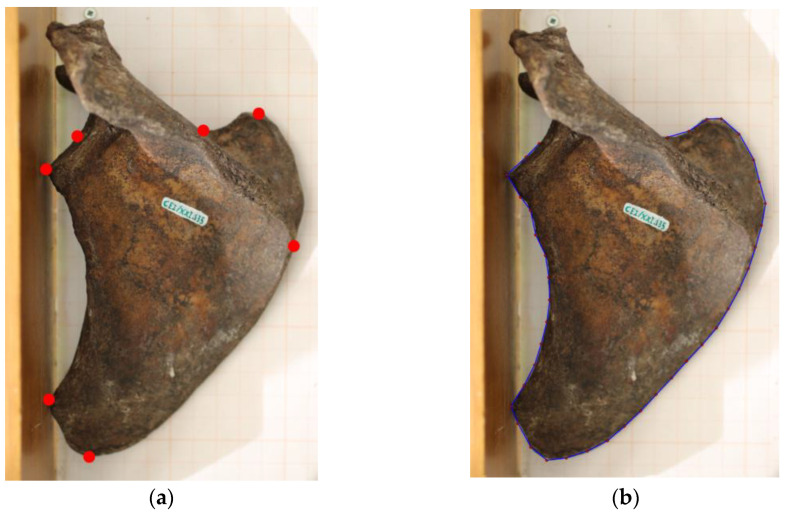
Representation of the landmarks (**a**) and semilandmarks (**b**) used in this study. The scapula was positioned on the posterior view, and both landmarks and semilandmarks were digitized.

The images were transferred to a workstation in order to place seven homologous landmarks in each scapula. The landmark set was based on previous research by Taylor and Slice [50] and Scholtz et al. [41]. While ignoring both the acromion and the spine, the landmarks mimic the shape of the scapular body and are clearly recognizable (Figure 1a):

Landmark 1: The medium point of the glenoid fossa, on the posterior point of the cavity.

Landmark 2: The point where the glenoid fossa touches the vertical surface of the osteometric board.

Landmark 3: At the position where the lateral border touches the vertical surface of the osteometric board.

Landmark 4: On the most inferior point of the inferior angle.

Landmark 5: Point of intersection of the scapular spine and the medial surface. The spine was followed until the point at which it would reach the medial border, considering that sometimes it splits and forms a triangular area.

Landmark 6: On the most superior point of the superior angle.

Landmark 7: Point of intersection of the scapular spine and the superior border. The point of intersection is found by following the superior border until it encounters the scapular spine. Due to individual variation, the scapular spine sometimes does not intersect with the superior border. In those cases, the point was recorded on the basis of the scapular notch.

All data were assembled with the *tps* software suite. Landmark digitation was accomplished through *tpsDig*. A scale was established at 1 cm, and the points were digitized from landmarks 1 through 7 in the same order.

Semilandmarks were also collected through *tpsDig*, using the function “Draw background curve”, allowing the drawing of the contour of each scapula, starting on landmark 1 and culminating on landmark 7. Subsequently, the function “Resample curve” was implemented with 40 points. The scale was also fixed to 1 cm (Figure 1b).

Data analysis was executed with MorphoJ [51] and PAST [52]. All GM procedures begin with a Procrustes Superimposition, or General Procrustes Analyses (GPA), that removes size, location, and orientation data to minimize the sum of squared distances between the homologous landmarks [53,54,55]. The resultant Procrustes shape coordinates only comprise shape information [44,56,57]. After the GPA, a Principal Components Analysis (PCA) was performed. The PCA can explore the key features of shape variation in a sample and ordinate the individuals in morphospace [51], allowing the extraction and evaluation of the main patterns of shape variation [55], simplifying and reducing the complexity of the data [44,54,58]. The comparison of the variation within groups with variation between groups was enacted through a Procrustes ANOVA (a permutation-based ANOVA) using the Procrustes coordinates [59]. Lastly, a regression was performed to evaluate the effects of the years of birth and death on scapular size and shape [27].

Intra-observer and interobserver errors were quantified in a previous study [49], with the results suggesting that errors are negligible.

## 3. Results

### 3.1. Landmarks Data

**Figure 2:**
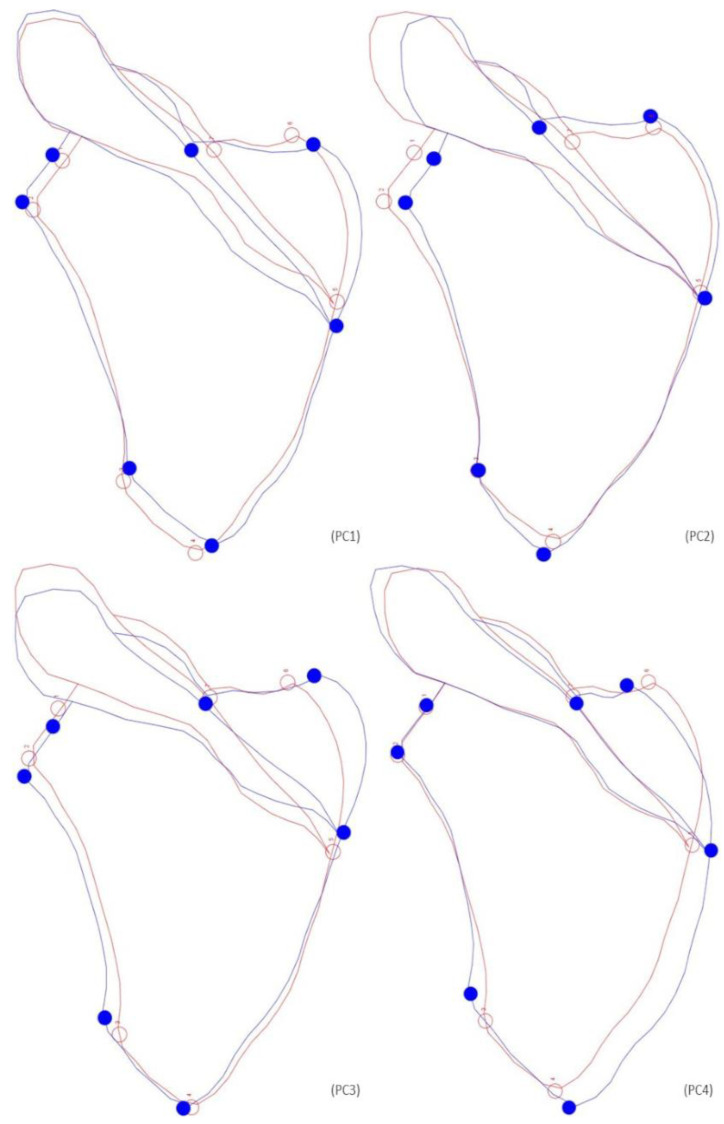
Graphic representation of the shape changes associated with the PCs in females, the starting shape is depicted as a red outline with hollow dots (at the position of the landmarks), while the target shape is shown as a blue outline with solid dots (at the position of the landmarks). PC1 represents an enlargement of the scapular body. PC2 demonstrates a small increase in length and a small straightening of the upper half of the lateral border. PC3 indicates a modest enlargement observed on the lateral and the upper part of the medial borders. PC4 shows an enlargement on the medial border.

A Procrustes ANOVA was used to evaluate the size and shape biological differences between individuals of the two identified skeletal collections. Analyses were performed within the confines of each biological sex. Concerning the females, only significant differences in shape were found (Table 4). Shape differences are accentuated on the lateral and medial surfaces, with an inferior angle more curved and acute in the CEI/XXI individuals. The pattern of shape variation can be explained by the first four Principal Components, or PCs (PC1—34.33%; PC2—24.19%; PC3—13.31%; PC4—11.07%), which accounted for 82.90% of the total shape variation (Figure 2). PC1 represents an enlargement of the scapular body, observed on both lateral and medial sides, as the length is slightly diminished. PC2 demonstrates a modest length increase on both superior and inferior surfaces and a small straightening of the upper lateral surface. For PC3, an enlargement of the scapular body in all the lateral borders and the upper part of the medial surface can be observed. PC4 shows an enlargement of almost the entire medial border and a slight length increase in the inferior angle. Nonetheless, substantial overlap in shape variability between the individuals of both collections can be observed. Size does not show a statistically significant influence on shape. The year of birth showed a significant effect on shape, being responsible for 2.24% of total shape variation. The same impact was observed for the year of death, influencing 2.52% of shape. The years of birth or death did not significantly influence scapular size (Table 5).

**Figure 3:**
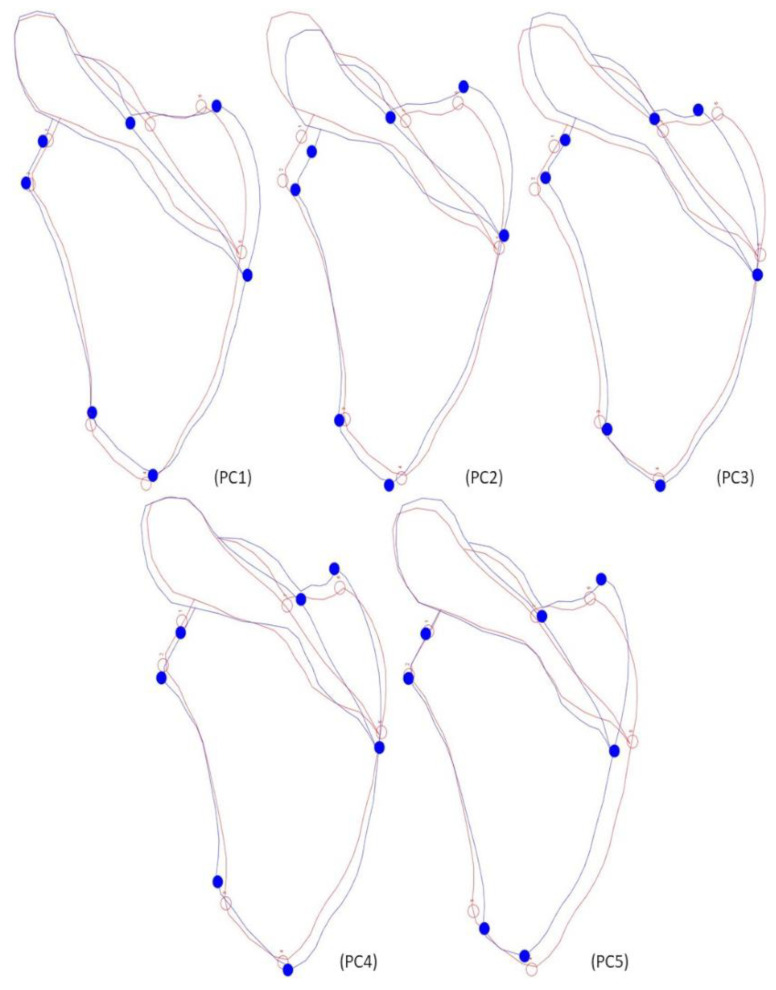
Graphic representation of the shape changes associated with the PCs in males. The starting shape is depicted as a red outline with hollow dots (at the position of the landmarks), while the target shape is shown as a blue outline with solid dots (at the position of the landmarks). PC1 enlargement of the scapular body, especially on the medial border. PC2 increases in length and on the upper medial border, with straightening observed on the glenoid fossa. PC3 straights on the lateral border and on the upper part of the medial border, the remaining medial surface enlarges. PC4 shows a relative increase in length and enlarges on the lower half of the medial border. PC5 straights on the medial border and increases its length on the superior angle.

**Figure 4:**
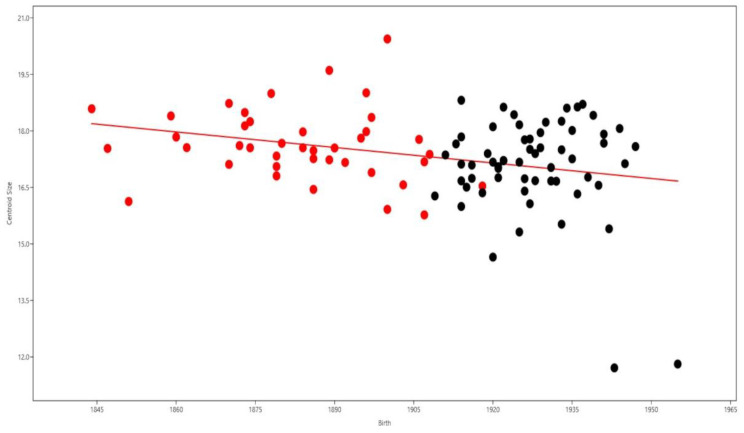
Comparison of the effect of birth years on scapular size; red dots represent CISC males, and black dots represent males from the CEI/XXI. The red line represents the evolution of size over time.

The Procrustes ANOVA suggests that the CISC and CEI/XXI males have significant biological differences in both size and shape (Table 6). The scapulae from the CISC males are generally larger. The medial and lateral borders of CEI/XXI individuals are more curved, and the inferior angle is more acute. The first five PCs described 88.79% of total shape variation (Figure 3). PC1 (31.16%) shows an enlargement of the body of the scapula for both surfaces and a small shortening of the length for the inferior border. PC2 (24.17%) depicts an increase in the length of the scapular body and a marginal enlargement on both lower and upper lateral and medial borders, as well as a slight decrease on upper and lower lateral and medial surfaces. PC3 (13.19%) displays a width decrease for the lateral and upper medial borders; the remaining medial border shows a minimal enlargement and a slight increase in length can also be observed. PC4 (10.58%) is responsible for a small increase in length. Lastly, PC5 (9.63%) shows a small decrease in length and width, observed on the positioning of the inferior surface and lateral border, respectively. Notwithstanding, males of both collections share a major overlap in shape variability (Figure 3). Size does not statistically influence the shape. The influence of the year of birth on shape was statistically significant, being accountable for 3.11% of shape variation; likewise, the year of death also showed a statistically significant effect on shape, influencing 3.73% of the total shape variation. Furthermore, the year of birth showed a negative association with size, influencing 8.20% of size variation (Figure 4). The year of death affected 6.29% of size variation (Table 5).

### 3.2. Semilandmarks Data

**Figure 5:**
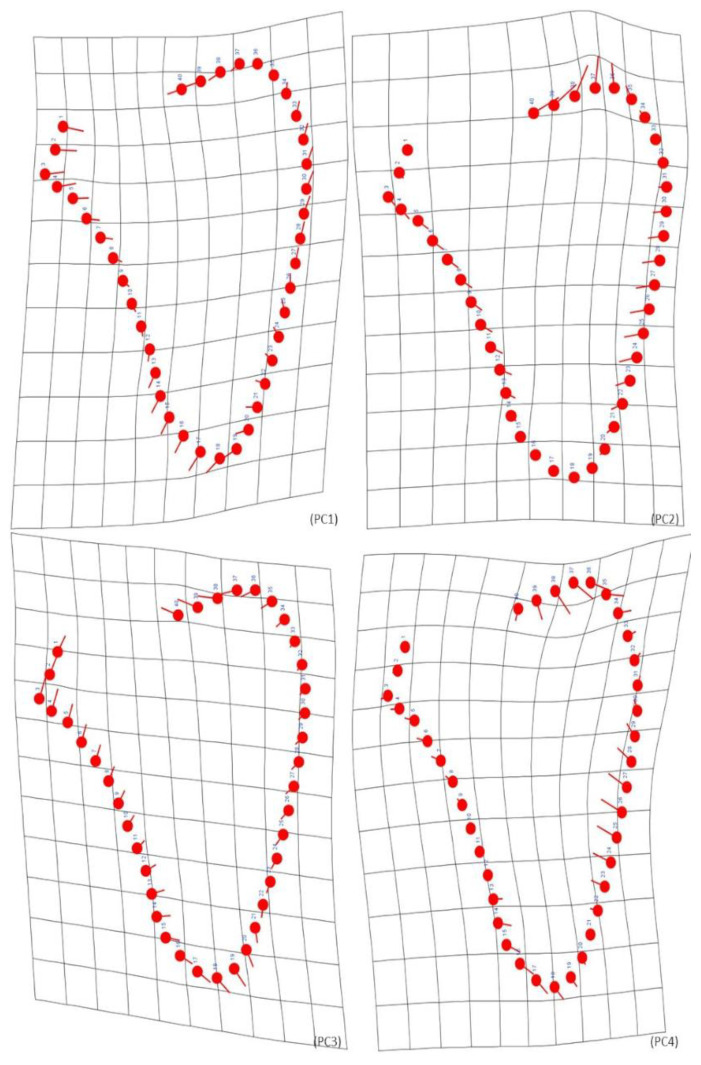
Graphic representation of the most significant PCs from semilandmarks’ data of female individuals. PC1 straights near the glenoid fossa and enlarges on the lower part of the lateral border, including a length increases on the inferior border. PC2 demonstrates an increase in length on the superior angle. PC3 indicates a small straightening on both surfaces, and the length increases on the inferior border. PC4 straights on the medial border.

The Procrustes ANOVA showed that female individuals exhibit significant differences in shape (Table 7). The scapular shape differences were manifest in the curvature of both the lateral and the upper part of the medial surface, being more accentuated in the CISC females. The superior angle was more prominent in CEI/XXI individuals. The patterns of shape variation can be explained by the first four PCs (Figure 5), which contain 82.39% of total shape variation (PC1—39.45%; PC2—22.04%; PC3—13.62%; PC4—7.28%). PC1 shows a shrinkage on the glenoid fossa area, the lower half of the medial border, and the superior surface. In the lateral surface, an increase in the length was noted until the lower part of the border, where it enlarges. In the remaining medial border, there is a small increase in length. PC2 also shows a decrease in width for the glenoid fossa area and both lateral and medial borders. In the superior border, an expansion in length was observed. PC3 reveals the reduction of width for the lateral, medial, and superior surfaces. In the glenoid fossa, the upper part of the lateral border, and the inferior surface, an increase in length was observed. Finally, PC4 shows a slight enlargement in the glenoid fossa and the upper area of the adjacent lateral border. Size does not seem to influence the shape. Both the years of birth and death impacted only 1.54% and 1.82% of the total shape variation, respectively (Table 5).

**Figure 6:**
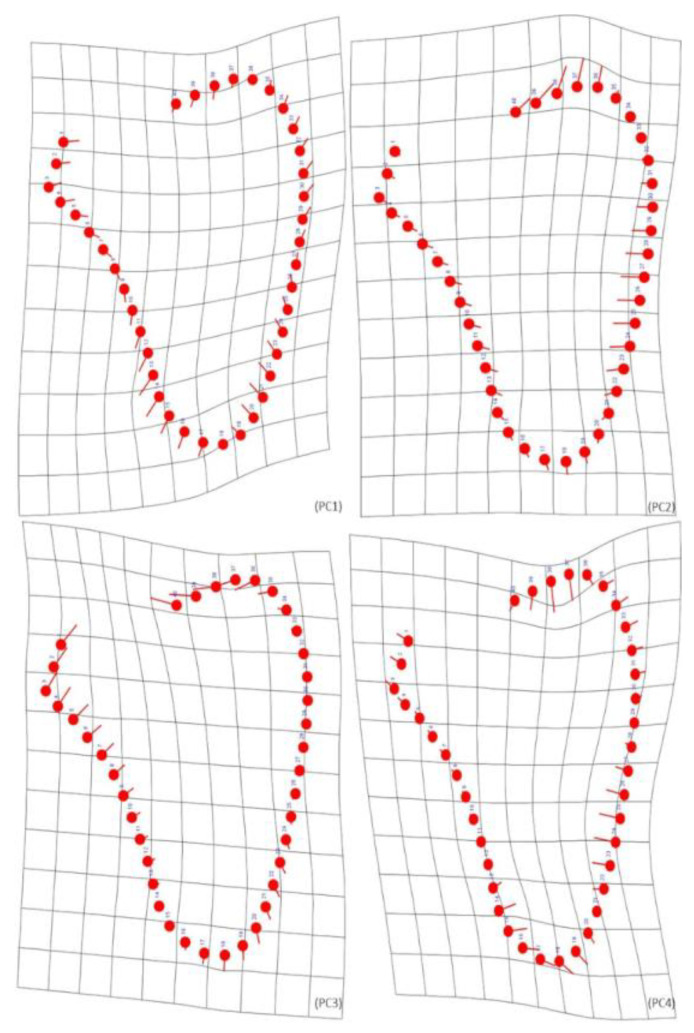
Graphic representation of the most significant PCs from semilandmarks’ data of male individuals. PC1 shows a significant enlargement on the lower half of the lateral border and superior part of the medial surface, the remaining medial border straights. PC2 expands the length on both superior and inferior surfaces, and the medial border suffers straights. PC3 increases in length on the inferior border and suffers a small straightening on the upper half of the lateral surface. PC4 diminishes in length on the superior border and increases near the inferior angle and straights on the middle of the medial border.

Both size and shape were statistically different between the CISC and CEI/XXI males (Table 8). The CISC individuals presented with larger scapulae. CEI/XXI males showed more accentuated curves in both medial and lateral, as the inferior and superior angles were more prominent. The results of the PCA showed that the first four PCs (PC1—58.06; PC2—17.94%; PC3—7.51%; PC4—4.51%) were responsible for 88.02% of the total pattern of shape variation (Figure 6). PC1 shows a slight decrease in width on the glenoid fossa and the lower area of the medial surface. On the lateral border, an increase in length can be observed, and in its lower area, an enlargement occurs; the same can happen in the upper medial surface. PC2 represents a decrease in the width of the scapular body, observed in the lateral and medial borders, and an increase in the length of both the superior and inferior surfaces. PC3 shows another decrease in the width of the lateral and superior borders. Finally, PC4 describes an increase in the width of both the glenoid fossa and the upper area of the medial border. The year of birth did not significantly influence shape variation. In contrast, the year of death was responsible for 4.49% of total shape variation (Table 5).

## 4. Discussion

This study suggests the presence of secular changes over a relatively short period of time in both the shape and size of the scapula in Portuguese nationals—particularly in males—between the late 19th and early 21st centuries. Secular trends refer to variation among individuals within a population that is justified primarily by differences in birth dates [60]. However, in order to assess secular trends and changing political and socio-economic circumstances in late-life living conditions, an examination of cohorts based on the year of death can also be of significance [61]. In females, the chronological trend is only detected for shape, with marginal changes associated with both the years of birth and death, suggesting a diachronic stability in the anatomy of the female scapula. In male individuals, a negative trend affecting the size of the scapula is highlighted. The observed scapular size decline in males with time opposes the general worldwide positive trend in stature and length of long bones, such as the radius, femur, and tibia, although these trends are neither universal nor show the same rates in all populations [8,18,20,25,29,62,63,64,65,66]. In fact, it follows the negative trend observed for measurements in the clavicle, humerus, pelvis, and cranial vault [5,8,20,24,31].

The dwindling of environmental growth-inhibiting factors began only during the 19th century when the living conditions of the population were enhanced through sociopolitical and technological advances, including improvements in sanitation, nutrition, and caloric intake, and the development of public health politics that conducted to the decrease of epidemic diseases while reducing infant mortality [8,18]. In Portugal, the scenario was reasonably different, as the effects of improving living conditions were only observable after the 1970s. The 19th century in Portugal was a tumultuous period characterized by the dearth of medical assistance and social policies, poor nutritional habits, inadequate sanitarian infrastructures, and epidemic diseases, where the base of the economy was largely agriculture-dependent [67,68].

It was only at the end of the century and at the beginning of the 20th century that the first public health measures were successful in decelerating epidemic conditions, slowly diminishing the mortality rate. Nonetheless, unquestionable improvements in living conditions only took place during the 1960s, especially after the creation of the Serviço Nacional de Saúde (National Health Service) in the wake of the Carnation Revolution that established a democratic regime in 1974 [62]. Portugal was then the European country with the highest percentage of young people and the lowest proportion of elderly people, the lowest life expectancy at birth, and the highest rates of fertility and infant mortality [62,63]. Currently, the population growth rate is negligible, and life expectancy has risen to average European values. Infant mortality declined, and it is among the lowest in the world (data available at https://www.pordata.pt/tema/europa/populacao-25, accessed on 24 May 2023). These data reflect the overall upgrading of the living conditions of the Portuguese population, dietary betterment, and the application of several health policies, such as the national vaccination plan and universal healthcare system [62,63,69].

Although the general improvement in the living conditions of the Portuguese population began later than in other European countries, a perceived positive increment in height ensued since the beginning of the 20th century [62,63]. In 1904, the mean height for 18-year-old males was higher in the Santarém district when compared to the Coimbra district [62,63,69]. The tendency to a taller but narrower body, the CEI/XXI males, born between 1910 and 1938, may have been affected by this trend, contrary to the CISC men who were previously born.

The improvement and stabilization of environmental and nutritional factors also impacted the age of menarche, which has been decreasing in the last decades [2,3]. Changes in sexual maturation influence skeletal maturation, leading to changes in adult morphology. Long bones became more linear, narrow, and gracile, and the distal long bones have increased more in length than the proximal long bones [8,20]. The clavicle has also been affected, with a decrease in its maximum length [31]. The pelvic morphology has also been influenced, with contemporary females and males presenting a more gracile pelvis when compared to chronologically ancient individuals [24].

Although there is a positive trend in stature, the human skeleton is veering towards a narrower form [8,24,25,31]. Narrower bodies are mostly connected to the rising standards of living and have been identified, for example, through the secular decrease of the average shoulder bi-deltoid breadth in men [70]. Also, the diachronic length reduction in the humerus was only observed in male individuals [20]. The results observed in the size of the scapulae in males seem to mimic these patterns. Hallgrímsson, Willmore, and Hall [71] theorize that different phenotypes can accumulate genetic variation that remains unexpressed if the environmental context remains stable. Once the population experience changes in the environment, the accumulated genetic variation might start to be expressed. Bipedality exempted the human upper limb from locomotive functions, and the recent mechanization of work has reduced most of their exertion of effort and strain, thus, conceivably increasing the susceptibility to secular changes, a trend that has been, in fact, observed [20,29]. The size of the clavicle has also been declining within a diachronic frame. Langley and Cridlin [31] suggest that the changes in the length of the clavicle are associated with an increase in body mass. The increase in the body mass index can be related to reduced physical activity, earlier skeletal maturation, or the intake of more nutritive food with diminished caloric expenditures [8,31].

The scapula articulates with the head of the humerus and the clavicle, forming the shoulder girdle. The actions performed by the shoulder girdle, such as moving the arm, are possible because most of the sixteen muscles associated are inserted on the scapula [72]. It is possible that the robusticity of the muscles of the shoulder declined in association with the declining size of both the clavicle and humerus. This influence, due to biomechanical forces, might be impacting the size of the scapula. Biomechanical forces are known factors for phenotypic alterations; for example, the robusticity of the masseter, temporal, and pterygoid muscles are decreasing, leading to a more gracile mandible [5,73]. These tendencies might vary in the same population; for example, males are more affected than females in this study, as observed by Jantz and Jantz [20] for the humerus and Spradley, Stull, and Hefner [74] for the cranium.

Different results were observed between the Procrustes ANOVA and regressions. The Procrustes ANOVA compared shape data from the sample classified into two pre-defined groups, an artificial classification, while regressions use data derived from the Procrustes coordinates and centroid size. This group pre-definition can exacerbate or obfuscate the differences related to secular changes observed in shape. In fact, there is some overlap—insignificant but nonetheless present—in the birth period of individuals of both collections. Regarding the techniques, there were also differences between landmarks and semilandmarks. This can be explained by their definition and the type of bone analyzed. Landmarks cannot evaluate surfaces or curves because they are not homologous points between the specimens [43]. Semilandmarks also offer valuable shape information contained on curves and surfaces [38,43,44]. The scapula is a flat triangular bone that has a great surface area with minimal points or protuberances [72,75]; therefore, the application of semilandmarks improved the attained shape information.

## 5. Conclusions

Secular changes in stature and age at menarche have concentrated most of the interest in the galaxy of anthropometric studies, with a dearth of analyses focused on the human skeleton, particularly in bones such as the scapula. This study benefited from an ample chronological, biological, and social variation expressed in two Portuguese skeletal identified collections to assess secular trends in the size and shape of the scapula through geometric morphometrics. Also, it was possible to evaluate size and shape variation independently. Both the size and shape of the scapula were affected through a moderately short period of time, with a particular evident tendency of scapular size decrement among males that possibly reflects general trends of the overall anatomy towards a narrower body. In females, the scapula presents, to an extent, with a morphological stasis, with a negligible time-variation of shape and none of size.

## Figures and Tables

**Table 1:** Distribution of individuals from both collections grouped by sex and age category.

Age Group (Years)	CEI/XXI		CISC		Number of Individuals	Percentage
	Females	Males	Females	Males		
17–29	0	0	6	5	11	5.21%
30–39	1	0	5	7	13	6.16%
40–49	0	0	10	9	19	9.00%
50–59	0	1	6	10	17	8.06%
60–69	5	11	7	5	28	13.27%
70–79	16	17	2	2	37	17.54%
80+	49	31	4	2	86	40.76%
Total	71	60	40	40	211	100%

**Table 2:** Distribution of individuals grouped by birth period, collection, and sex.

Birth Period (Years)	CEI/XXI		CISC		Number of Individuals	Percentage
	Females	Males	Females	Males		
1820–1844	0	0	3	1	4	1.90%
1845–1869	0	0	9	5	14	6.64%
1870–1894	0	0	17	21	38	18.01%
1895–1919	22	13	11	13	59	27.96%
1920–1944	47	44	0	0	91	43.13%
1945–1969	1	3	0	0	4	1.90%
1970+	1	0	0	0	1	0.47%
Total	71	60	40	40	211	100%

**Table 3:** Distribution of individuals grouped by death period, collection, and sex.

Death Period (Years)	CEI/XXI		CISC		Number of Individuals	Percentage
	Females	Males	Females	Males		
1910–1929	0	0	12	9	21	9.95%
1930–1949	0	0	28	31	59	27.96%
1950–1969	0	0	0	0	0	0%
1970–1989	0	0	0	0	0	0%
1990–2009	65	50	0	0	115	54.50%
2010+	6	10	0	0	16	7.58%
Total	71	60	40	40	211	100%

**Table 4:** Procrustes ANOVA results based on landmark data revealing significant shape differences between females from the CISC and CEI/XXI.

Centroid Size					
Effect	SS	MS	df	F	P (param.)
Collection	6.186875	6.186875	1	2.81	0.0965
Residual	239.907978	2.200991	109		
Shape					
Effect	SS	MS	df	F	P (param.)
Collection	0.01425634	0.001425634	10	2.42	0.0076
Residual	0.64262408	0.000589563	1090		

SS—sum of squares; MS—mean squares.

**Table 5:** Results of the regressions performed for both sexes regarding landmark and semilandmark data. We tested the impact of size on the shape and the influence of the birth and death periods on both shape and size. P-V represents the *p*-value; only *p* < 0.05 are considered statistically significant. %P represents the percentage of the influence on total shape or size variation.

Impacts	Females				Males			
	Landmarks		Semilandmarks		Landmarks		Semilandmarks	
	P-V	%P	P-V	%P	P-V	%P	P-V	%P
Size-Shape	0.0789	1.79%	0.2069	1.29%	0.1534	1.60%	0.1665	1.67%
Birth-Shape	0.0346	2.24%	0.1319	1.54%	0.0070	3.11%	0.0752	2.42%
Death-Shape	0.0199	2.52%	0.0811	1.82%	0.0025	3.73%	0.0098	4.49%
Birth-Size	0.0514	3.48%	0.1886	1.56%	0.0047	8.20%	0.0008	11.31%
Death-Size	0.0915	2.46%	0.3288	0.92%	0.0087	6.29%	0.0010	10.44%

P-V—*p*-value; %P—% Predicted.

**Table 6:** Procrustes ANOVA results based on landmark data reveal significant differences in both size and shape between males from CISC and CEI/XXI.

Centroid Size					
Effect	SS	MS	df	F	P (param.)
Collection	8.390224	8.390224	1	5.85	0.0174
Residual	140.577483	1.434464	98		
Shape					
Effect	SS	MS	df	F	P (param.)
Collection	0.02360341	0.002360341	10	4.14	<0.0001
Residual	0.55808035	0.00056947	980		

SS—sum of squares; MS—mean squares.

**Table 7:** Procrustes ANOVA results based on semilandmarks’ data reveal significant shape differences between females from the CISC and CEI/XXI.

Centroid Size					
Effect	SS	MS	df	F	P (param.)
Collection	709,694.3831	709,694.3831	1	1.03	0.3119
Residual	74,942,576.75	687,546.5757	109		
Shape					
Effect	SS	MS	df	F	P (param.)
Collection	0.00808474	0.000106378	76	1.77	<0.0001
Residual	0.49710356	6.00 × 10^−5^	8284		

SS—sum of squares; MS—mean squares.

**Table 8:** Procrustes ANOVA results based on semilandmarks’ data reveal significant differences in both size and shape between males from CISC and CEI/XXI.

Centroid Size					
Effect	SS	MS	df	F	P (param.)
Collection	61.841885	61.841885	1	10.24	0.0019
Residual	591.970676	6.040517	98		
Shape					
Effect	SS	MS	df	F	P (param.)
Collection	0.03121162	0.000410679	76	5.49	<0.0001
Residual	0.55739811	7.48386 × 10^−5^	7448		

SS—sum of squares; MS—mean squares.

## Data Availability

Data are available upon reasonable request.
